# Edible Insect-Based Beverages: A Narrative Review of Functional, Technological, and Experimental Dimensions

**DOI:** 10.3390/insects17040384

**Published:** 2026-04-02

**Authors:** Oscar Abel Sánchez-Velázquez, Alan Javier Hernández-Álvarez, Luis Mojica

**Affiliations:** 1 School of Food Science & Nutrition, University of Leeds, Woodhouse Lane, Leeds LS2 9JT, UK; a.j.hernandezalvarez@leeds.ac.uk; 2 National Alternative Protein Innovation Centre (NAPIC), UK; 3 Unidad de Tecnología Alimentaria, Centro de Investigación y Asistencia en Tecnología y Diseño del Estado de Jalisco, A.C. (CIATEJ), Zapopan 45019, Mexico; lmojica@ciatej.mx

**Keywords:** entomophagy, functional beverages, experiential foods, traditional beverages, consumer acceptance, food innovation, culinary storytelling

## Abstract

Edible insects are increasingly recognized as sustainable and nutrient-dense ingredients with potential applications across diverse food systems. While their use in solid foods has been widely explored, the incorporation of insect-derived ingredients into beverages remains fragmented and insufficiently conceptualized. This narrative review critically examines the current state of insect-based beverages, integrating technological, nutritional, cultural, and market-oriented perspectives. Rather than adopting a fully systematic review methodology, this article synthesizes representative scientific literature, traditional practices, and emerging commercial examples to explore how edible insects are being positioned within two distinct yet complementary pathways: (i) functional beverages targeting nutrition, gut health, sports performance, immunity, and meal replacement, and (ii) experiential beverages driven by culinary storytelling, tradition, ritual, and sensory innovation, such as insect-infused beers and spirits. Relevant sources were identified through searches of major scientific databases using combinations of keywords such as edible insects, entomophagy, insect-based beverages, functional beverages, and insect protein, with emphasis on peer-reviewed literature published mainly over the past two decades, complemented by representative examples of traditional practices and emerging commercial products. The review discusses the nutritional and biofunctional potential of insect-derived proteins, lipids, and chitin-related components, with particular emphasis on their behavior in liquid matrices, including solubility, stability, flavor impact, and processing constraints. Technological challenges associated with beverage formulations are critically assessed. In parallel, consumer perception is examined through the lens of neophobia, sensory expectations, and the role of cultural framing and gastronomy in facilitating acceptance. By bridging food science, beverage technology, and food culture, this review identifies key knowledge gaps and research priorities for advancing insect-based beverages beyond niche applications. It argues that future development in this field will depend not only on technological optimization and a regulatory framework, but also on the deliberate design of products that integrate functionality with meaningful culinary and cultural narratives.

## Simple Summary

Edible insects are increasingly explored as sustainable and nutritious food ingredients, but their use in beverages has received far less attention than in solid foods. Beverages present unique challenges, such as maintaining stability, appearance, and pleasant taste, which make the incorporation of insect-derived ingredients more complex. This review examines how insects are currently used in drinks, combining scientific studies, traditional practices, and emerging commercial products. Two main approaches are identified. One focuses on functional beverages, such as protein shakes or fermented drinks, in which insects are processed to provide nutritional benefits, such as high-quality protein or support for gut health. The other emphasizes experiential beverages, including beers, spirits, and traditional drinks, where insects contribute to flavor, cultural meaning, or storytelling rather than nutrition. The review highlights key technological challenges, such as ingredient solubility and flavor control, as well as social factors like consumer acceptance and cultural context. In summary, this work shows that insect-based beverages are likely to move beyond niche markets when technological development is combined with clear product design, cultural understanding, and transparent communication, helping society explore more sustainable and diverse food options.

## 1. Introduction

The growing pressure on global food systems to deliver nutritionally adequate, environmentally sustainable, and socially acceptable foods has intensified interest in alternative ingredients, including edible insects. Insects are widely recognized for their high-quality protein content, favorable amino acid profiles, essential fatty acids, micronutrients, and comparatively low environmental footprint when contrasted with conventional livestock [1,2]. Nevertheless, recent analyses suggest that sustainability advantages may vary depending on farming practices, feed substrates, and processing requirements, indicating that environmental benefits should be evaluated within specific production contexts rather than assumed to be universal. Consequently, research on edible insects for human consumption has expanded substantially over the last decade, particularly in relation to solid food applications such as bakery products, snacks, protein-enriched foods, and meat analogues [3,4].

In contrast, the application of edible insects in beverages remains limited and conceptually fragmented. Beverage matrices present specific technological, sensory, and regulatory challenges that differ fundamentally from those associated with solid foods. Parameters such as protein solubility under acidic conditions, sedimentation, visual appearance, aroma release, mouthfeel, and microbial stability are critical determinants of beverage quality and cannot be directly extrapolated from solid food systems [5,6]. Nevertheless, insect-derived ingredients are increasingly being explored for use in protein shakes, smoothies, fermented beverages, beers, spirits, teas, and experimental milk analogues, both in scientific studies and niche commercial products [7,8,9].

Beyond technological considerations, insect-based beverages occupy a distinctive intersection between nutrition, gastronomy, and cultural meaning. While many contemporary product concepts emphasize sustainability and functional benefits, several traditional and emerging beverages incorporating insects are consumed not primarily for nutritional optimization, but for their sensory attributes, symbolic value, and experiential dimensions. Traditional examples such as mezcal containing maguey worms illustrate how insects can serve as culinary agents embedded in rituals, narratives, and place-based food identities rather than merely as nutrient sources [9,10].

Despite the diversity of existing applications, the current literature lacks an integrative framework to link the nutritional and techno-functional potential of edible insects with their cultural, sensory, and experiential roles in beverage systems. Previous reviews have often generalized insect applications across food categories or presented catalogues of isolated examples without sufficient critical synthesis. Moreover, methodological expectations associated with systematic or comprehensive reviews may be ill-suited to an emerging and heterogeneous field characterized by a combination of experimental research, traditional practices, and early-stage market innovation.

Therefore, the objective of this narrative review is to critically examine the current state of insect-based beverages by synthesizing representative scientific literature, traditional knowledge, and commercial developments already reported in the field. Rather than aiming for exhaustive coverage, this review seeks to clarify how edible insects are currently positioned within beverage systems, identify key technological and conceptual challenges, and highlight knowledge gaps that must be addressed to enable further development. To support this narrative synthesis, relevant literature was identified through searches in major scientific databases, including Scopus (https://www.scopus.com), Web of Science (https://www.webofscience.com), and Google Scholar (https://scholar.google.com). Searches were conducted using combinations of keywords such as edible insects, entomophagy, insect-based beverages, functional beverages, insect protein, and insect fermentation. The selection focused primarily on peer-reviewed articles and review papers published over the past two decades, complemented by representative examples of traditional practices and emerging commercial products when relevant to beverage applications. To this end, the review adopts a conceptual framework that distinguishes between two complementary yet distinct pathways in the evolution of insect-based beverages: functional beverages and experiential, storytelling-driven beverages.

## 2. Conceptual Framework: Two Pathways for Insect-Based Beverages

**Figure 1:**
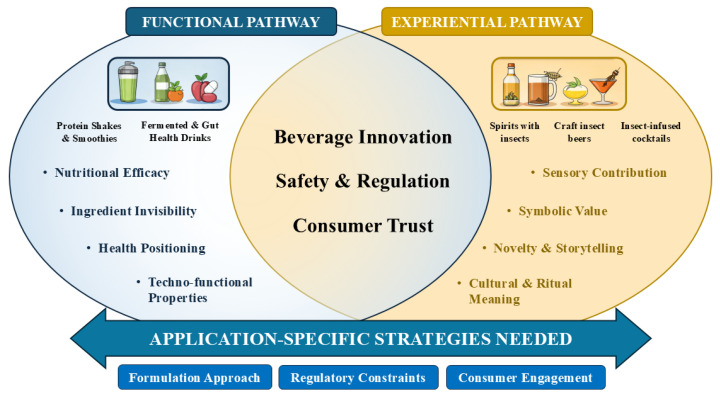
Conceptual framework illustrating the two partially overlapping pathways for the development of insect-based beverages.

The development of insect-based beverages does not follow a single, linear trajectory. Instead, existing research, traditional practices, and commercial examples suggest the emergence of two partially overlapping pathways that differ in their primary objectives, design logic, and value propositions. Recognizing this distinction is essential for understanding both the opportunities and the limitations of edible insects in beverage applications. Based on the analysis of current research, traditional practices, and commercial developments, insect-based beverages can be conceptually organized into two partially overlapping pathways: functional beverages and experiential, storytelling-driven beverages (Figure 1).

### 2.1. Functional Insect-Based Beverages

The first pathway positions edible insects primarily as functional ingredients. In this context, insects are valued for their nutritional density and biofunctional components, including proteins, peptides, lipids, minerals, and chitin-related fractions [1,11]. Beverages developed within this pathway are designed to deliver specific physiological benefits, such as supporting muscle recovery, improving gut health, enhancing immune function, or serving as meal replacements [12,13].

Examples include protein shakes and smoothies enriched with insect powders or protein isolates, fermented beverages containing insect protein hydrolysates, chitin- or chitosan-enriched drinks aimed at modulating gut microbiota, and recovery or energy drinks formulated for physically active consumers [7,14,15]. In these products, insects are typically processed into refined fractions to minimize sensory impact and improve compatibility with liquid matrices. Processing strategies such as enzymatic hydrolysis, fermentation, ultrasonication, and deodorization are often employed to enhance solubility, digestibility, and consumer acceptance [2,6,8].

Within this pathway, technological performance and scientific validation are central. Key challenges include achieving adequate protein solubility at beverage-relevant pH values, preventing sedimentation, managing color and aroma, ensuring microbiological safety, and complying with regulatory frameworks governing novel foods [16,17]. Consumer acceptance strategies frequently depend on familiar beverage formats and health-oriented positioning, with insect-derived ingredients rendered invisible or sensorially neutral within the formulation.

### 2.2. Experiential and Storytelling-Driven Beverages

The second pathway emphasizes the experiential, culinary, and cultural dimensions of insect-based beverages. In this case, insects are not incorporated primarily to optimize nutritional profiles, but to contribute flavor, texture, symbolism, authenticity, or narrative value. Consumption is framed as an experience rather than as a functional or nutritional alternative.

Traditional beverages such as mezcal containing maguey worms (Lepidoptera larvae) exemplify this pathway, where the insect serves as a marker of identity, ritual, and artisanal heritage [9,18]. Similarly, craft beers brewed with insects, insect-infused spirits, and cocktails featuring insect-derived garnishes or salts leverage novelty, sensory complexity, and storytelling to engage consumers [8,19]. In these cases, the insect is often visible or explicitly highlighted, and its presence is closely linked to notions of tradition, terroir, and culinary exploration.

This pathway aligns with broader perspectives on food acceptance that emphasize the role of context, preparation, presentation, and social meaning in shaping consumer responses to novel foods [20]. Rather than attempting to conceal insects, experiential beverages integrate them into coherent culinary narratives involving rituals, chefs, serving practices, and culturally grounded storytelling.

From a scientific standpoint, this pathway raises distinct questions. Sensory contribution, flavor chemistry, stability during fermentation or aging, and interactions with other ingredients become central considerations, while nutritional optimization may be secondary. Regulatory challenges related to labeling, species identification, and consumer information are particularly relevant given the visibility of insects in these products [17].

### 2.3. Complementarity Rather than Opposition

Importantly, these two pathways should not be interpreted as mutually exclusive. Hybrid products may combine functional objectives with experiential design, for example, by embedding insect-derived proteins within culturally meaningful beverage formats or by pairing performance-oriented drinks with strong sustainability narratives. However, failure to distinguish between these pathways can lead to conceptual ambiguity, superficial analysis, and unrealistic expectations regarding consumer acceptance.

By adopting this dual-pathway framework, this review provides a structured lens for critically examining the nutritional, techno-functional, sensory, cultural, and regulatory dimensions of insect-based beverages. The following sections build on this framework to assess current knowledge, identify limitations, and outline priorities for future research and product development.

This conceptual distinction does not imply rigid categories but rather analytical orientations that help interpret the diverse ways insects are incorporated into beverage systems. Functional beverages tend to prioritize nutritional efficacy, ingredient invisibility, and technological optimization, whereas experiential beverages emphasize sensory novelty, cultural narratives, and culinary storytelling. Recognizing these distinct design logics can help guide both research priorities and product development strategies.

## 3. Nutritional and Biofunctional Potential of Edible Insects in Beverage Matrices

Edible insects are widely recognized as nutrient-dense ingredients, providing high-quality proteins, essential amino acids, unsaturated fatty acids, vitamins, minerals, and structural polysaccharides such as chitin [1,21,22]. However, the nutritional relevance of these components in beverage applications cannot be inferred directly from their performance in solid foods. Liquid matrices impose specific constraints related to ingredient dispersion, stability, bioaccessibility, and sensory perception [23], which may significantly alter the nutritional effectiveness of insect-derived ingredients.

Proteins are the primary target fraction in beverage formulation, particularly in functional beverages such as protein shakes, recovery drinks, and meal replacement beverages [24]. Insects such as *Acheta domesticus*, *Tenebrio molitor*, and *Alphitobius diaperinus* can contain up to 50–70% protein on a dry matter basis, depending on species, life stage, and processing [21,25]. Their amino acid profiles are generally balanced and comparable to those of conventional animal proteins, with relevant levels of lysine, leucine, valine, and other essential amino acids associated with muscle protein synthesis and metabolic health [26,27].

From a beverage perspective, however, protein quality must be evaluated not only in terms of composition but also in terms of bioavailability and functional behavior under beverage-relevant conditions. Human intervention studies have shown that insect proteins can elicit postprandial aminoacidemia and muscle protein synthesis responses comparable to those of milk, whey, or beef proteins when consumed in isolated or concentrated forms [27,28,29]. These findings support the potential of insect proteins in sports and recovery beverages. However, their slower digestion kinetics suggest that they may function more effectively as sustained-release protein sources rather than fast-acting supplements. Equally, potential allergenicity should be considered, as certain insect proteins may cross-react with allergens in crustaceans and other arthropods, particularly in individuals with pre-existing shellfish allergies.

Beyond proteins, insect-derived lipids and micronutrients may contribute additional value to beverage formulations. Insects contain unsaturated fatty acids, including omega-3 and omega-6 fatty acids, as well as minerals such as iron, zinc, and calcium [26,30]. However, lipid fractions pose technological challenges in beverages due to oxidation, flavor instability, and phase separation [31]. As a result, lipid removal, or partial defatting, is often required when formulating beverages for neutral flavor profiles, particularly in functional applications.

Chitin and its derivatives constitute another biofunctional fraction relevant to beverages targeting gut health. Chitin contents in edible insects typically range from 3 to 12% of dry weight and function as insoluble dietary fiber [11]. Although native chitin has limited solubility, its partial hydrolysis into chitosan or chitooligosaccharides can enhance fermentability and prebiotic potential, making it suitable for gut-health-oriented beverages [32,33]. Nevertheless, the contribution of chitin-related compounds to beverage functionality depends strongly on processing, dosage, and interaction with the beverage matrix, highlighting the need for targeted formulation strategies.

Importantly, the nutritional contribution of edible insects may differ substantially between the functional and experiential beverage pathways. While functional beverages aim to deliver measurable physiological benefits through optimized formulations and defined dosages, experiential beverages such as beers, spirits, and traditional infusions often incorporate insects in quantities insufficient to generate nutritional effects. In these cases, nutritional claims may be secondary or irrelevant, and the value of insects lies primarily in their sensory or symbolic contribution.

## 4. Techno-Functional Challenges of Insect-Derived Ingredients in Liquid Systems

The successful incorporation of edible insects into beverages depends not only on their nutritional potential but also on their techno-functional performance in liquid systems [5,34,35,36]. Compared to solid foods, beverages impose stricter requirements related to solubility, dispersion, stability, visual appearance, aroma, and mouthfeel. Failure to address these parameters has been identified as a major barrier to the development and acceptance of insect-based beverages [3,16].

Protein solubility is one of the most critical challenges. Many functional beverages, including fruit-based drinks, fermented beverages, energy drinks, and isotonic formulations, operate at acidic pH values (typically pH 2.8–4.5). Under these conditions, insect proteins—particularly when used as whole flours—may exhibit limited solubility, leading to precipitation, sedimentation, and phase separation [5,36]. Protein concentrates and isolates generally display improved solubility and emulsifying properties compared to whole insect powders, making them more suitable for beverage applications [6,37]. For example, insect protein isolates and concentrates from Acheta domesticus and Gryllus assimilis have shown good water-holding capacity (1.1–4.2 g/g), oil-holding capacity (>48%), and emulsification stability/capacity (29–86%) [6,38]. These properties are beneficial for creating smooth-textured beverages such as protein shakes and smoothies. Additionally, insect proteins can contribute to beverage viscosity and stability, improving mouthfeel and overall sensory appeal [5].

Processing technologies play a central role in improving the techno-functional performance of insect-derived ingredients. Enzymatic hydrolysis, fermentation, ultrasonication, and membrane-based fractionation have been shown to enhance protein solubility, digestibility, and emulsifying capacity while reducing molecular weight and allergenic potential [7,19,39]. Fermentation, in particular, has demonstrated the ability to improve protein digestibility and bioavailability by 40–100% [40] while reducing allergenic potential [2,39,41] and maintaining probiotic viability in fermented beverages enriched with insect protein hydrolysates [14,42].

In addition to their nutritional and techno-functional properties, the potential allergenicity of insect-derived proteins must also be considered in beverage applications. Several studies have reported cross-reactivity between insect proteins and allergens from crustaceans, mites, and other arthropods, particularly tropomyosin and arginine kinase [2,39,41,42]. As a result, individuals with shellfish allergies may also be at risk of allergic reactions when consuming insect-based products. Processing methods—including thermal treatment, enzymatic hydrolysis, and fermentation—may partially modify allergenic proteins and reduce their immunoreactivity [41,42]; however, the extent of this reduction varies depending on the insect species, processing conditions, and final product formulation. These considerations highlight the importance of appropriate allergen labeling and consumer information when developing insect-based beverages and other food products.

Sensory attributes represent another major challenge. Edible insects are associated with characteristic aromas and flavors, often described as earthy, nutty, or roasted, which may be desirable in certain contexts but problematic in neutral or fruit-flavored beverages [8]. Lipid oxidation products and Maillard reaction compounds are key contributors to these sensory notes. Partial defatting, deodorization, and controlled fermentation can reduce off-flavors, while flavor-masking strategies using cocoa, vanilla, spices, or fruit extracts may further enhance acceptability [2,43].

Visual appearance and mouthfeel are particularly relevant in beverage systems, where consumers often expect homogeneity and clarity. Studies on traditional beverages such as “chucula” fortified with cricket flour (3%, 5%, and 7%) have shown that increasing insect inclusion levels can negatively affect color parameters (intensifying the reddish-brown tones of the original recipe) and visual acceptance, even when nutritional value is improved [44,45]. These findings underscore the importance of optimizing particle size, concentration, and processing methods to balance nutritional enhancement with sensory quality.

Notably, techno-functional requirements differ substantially between functional and experiential beverages. In functional drinks, invisibility, stability, and neutral sensory profiles are often prioritized. In contrast, experiential beverages such as beers, spirits, and traditional infusions may tolerate—or even value—turbidity, color changes, and distinctive flavors derived from insect ingredients [8,9]. This divergence reinforces the need for application-specific formulation strategies rather than a one-size-fits-all approach.

## 5. State of the Art of Insect-Based Beverages

The current landscape of insect-based beverages is characterized by heterogeneity in formulation strategies, technological maturity, and intended value propositions. Existing products and research efforts range from highly engineered functional beverages to traditional and experiential drinks in which insects play a symbolic or sensory role. Rather than representing a single coherent market, insect-based beverages are best understood as a collection of application-specific niches at different stages of development. Representative examples of experimental, commercial, and traditional insect-based beverages are summarized in Table 1, highlighting the insect species used, formulation approach, processing level, and primary value proposition.

The examples compiled in Table 1 illustrate that insect-derived ingredients are incorporated into beverages through distinct yet sometimes overlapping formulation logics that correspond broadly to the conceptual pathways proposed in this review. In the functional pathway, insects are typically processed into technologically compatible fractions—such as powders, protein concentrates, isolates, or hydrolysates—that can be integrated into beverages with the aim of delivering measurable nutritional or physiological benefits. In these cases, formulation strategies prioritize solubility, digestibility, and sensory neutrality, and the insect ingredient is often rendered visually or organoleptically unobtrusive within the final product. By contrast, in the experiential pathway, insects are frequently incorporated as whole organisms or minimally processed ingredients whose value lies less in nutritional optimization and more in their capacity to generate distinctive sensory attributes, cultural symbolism, or narrative value within the beverage. However, the boundary between these categories is not absolute. Certain products simultaneously draw on functional claims (e.g., protein enrichment) and experiential elements such as novelty, visibility, or culinary storytelling. This partial overlap suggests that the functional–experiential distinction should be interpreted as an analytical continuum rather than as a rigid classification system. Nevertheless, the examples summarized in Table 1 demonstrate that most current applications tend to cluster around one of these two dominant design logics, supporting the usefulness of this framework for interpreting the emerging landscape of insect-based beverages. This interpretation also highlights how technological development, cultural context, and product design interact in shaping the emerging category of insect-based beverages.

This section critically reviews the main beverage categories in which edible insects have been incorporated, distinguishing between experimental formulations, commercially available products, and traditional beverages. Emphasis is placed on formulation rationale, processing approaches, and the extent to which insects contribute functional, nutritional, or experiential value. Some commercial descriptions of these products (Table 1) attribute various medical, functional and/or experiential properties. However, these claims primarily originate in marketing narratives and are not supported by scientific evidence. In this context, their relevance lies in illustrating how experiential beverages are framed and communicated within commercial settings rather than in demonstrating verified health effects.

### 5.1. Protein Shakes and Smoothies

Protein shakes and smoothies represent the most technologically advanced and scientifically substantiated category of insect-based beverages. Within this segment, edible insects are primarily positioned as alternative protein sources targeting sports nutrition, recovery, and meal supplementation markets. Most formulations rely on powdered insect ingredients, protein concentrates, or isolates derived from species such as *Acheta domesticus*, *Tenebrio molitor*, and *Alphitobius diaperinus* [6,21].

Experimental and commercial products generally favor protein isolates or partially defatted powders over whole insect flours due to their improved solubility, emulsifying properties, and reduced sensory impact [5,36]. Human intervention studies support the suitability of insect proteins for this category, demonstrating postprandial amino acid availability and muscle protein synthesis responses comparable to conventional animal proteins [27,28,29]. For example, Dai et al. [29] reported that the immediate consumption of 25 g of cricket protein resulted in variations in post-meal plasma leucine levels until 1.21-fold at 90–180 min (*p* < 0.0001) vs. the control (25 g of beef protein), as well as in all essential amino acids (EAAs) until 1.16-fold at 90–180 min (*p* < 0.001). However, it had non-distinct impacts (*p* > 0.0001) on appetite hormones, appetite, and voluntary energy intake among young men. Vangsoe et al. [28] observed a significant increase (over 140%) in EAA blood levels within 120 min of consuming 25 g of lesser mealworm (*Alphitobius diaperinus*) protein (82% protein content) in six healthy young males. This increase was compared to whey and soy proteins. However, no significant changes (*p* > 0.05) were noted in branched-chain amino acid (BCAA) or leucine concentrations. Hermans et al. [27] investigated the effects of consuming a meal-sized portion of 30 g of protein derived from lesser mealworm, finding that it leads to quick protein digestion and amino acid absorption (73%), which in turn boosts muscle protein synthesis rates both at rest and during post-exercise recovery at 5 h. The postprandial processing of protein derived from lesser mealworms in the human body appears to be comparable to that of milk protein concentrate, with a reported rate of 0.015–0.073%/h. These findings indicate that the consumption of insect protein can result in circulating amino acid levels comparable to those observed following the intake of conventional animal-derived protein sources, such as milk protein. Still, it showed a trend for higher concentrations at 120 min or over, suggesting it may act as a “slow” digestible protein source. This highlights its potential as a sustained-release protein option for active individuals, especially given its balanced amino acid profile and the presence of BCAAs, which are essential for muscle recovery. However, to position insect-derived proteins as a viable alternative in the sports and functional nutrition market, there is now a need for reliable suppliers of high-quality protein concentrates and isolates, as well as effective flavor-masking technologies to enhance consumer acceptance.

From a formulation standpoint, insect protein shakes and smoothies often incorporate plant-based ingredients, such as fruits, seeds, and plant milks, to enhance flavor, mouthfeel, and consumer familiarity. However, several commercial products provide limited transparency regarding the proportion of insect protein relative to other protein sources, which complicates the assessment of their nutritional relevance. Moreover, many products rely on whole insect flours rather than purified fractions, potentially limiting solubility and increasing sensory variability.

Nowadays, it is possible to find various protein powder options containing edible insects, designed for protein shakes. These are some examples of powder made with house cricket (*Acheta domesticus*): Cricket powder by BugEater Foods (USA, https://www.bugeaterfoods.com); Chocolate Protein Powder by Mighty Cricket (USA, https://mightycricket.com); Vanilla Bean Cricket Protein Shake by Earth Proof (Canada, https://earthproofprotein.com); Natural Protein Powder by Circle Harvest (Australia, https://circleharvest.com.au); High Protein Cricket Powder (UK, https://www.bugvita.com); and Protein Powder by Gricha (Mexico, https://gricha.com). In many of these products, the specific content of insect protein is not indicated; however, its proportional representation within the formulation is typically second or third, frequently preceded by plant-based protein ingredients such as protein isolates or protein concentrates derived from rice, pea, or other grains. It is worth noting that when cricket is the predominant ingredient in those products, it is often included in whole-flour powder rather than as a protein concentrate or isolate. Purifying proteins could help remove off-flavors and reduce lipid calories, making cricket a more attractive source of amino acids. Although other insect species hold significant promise for creating powders for protein shakes, such as grasshoppers and locusts, they have not been investigated to the same extent as the house cricket.

Insect protein-based smoothies are emerging as a sustainable and nutritious alternative to traditional protein supplements. Insect proteins are highly digestible and offer favorable sensory attributes, including a mild, nutty flavor that complements a wide range of ingredients without overpowering them [8,53]. The high solubility and stability of insect proteins help maintain a smooth consistency, while the inclusion of fruits and seeds contributes essential nutrients such as vitamin C, fiber, and omega-3 fatty acids [3]. In addition, edible insect powders (e.g., *Acheta domesticus*) exhibit high emulsifying (>57%) and foaming (>80%) capacities, as well as stability, properties highly valued in the formulation of innovative smoothies. However, these functional attributes depend strongly on physicochemical factors such as particle size and pH [5,6].

Companies such as EXO Protein (https://exoprotein.com), Entomo Farms (https://entomofarms.com), and BugEater Foods (http://bugeaterfoods.com) are at the forefront of this trend, offering high-quality insect protein powders that are easily integrated into everyday smoothie recipes. Nevertheless, in the market, only a few commercial products are available, such as the Cricket Milkshake by Wayback Burgers© (USA, https://waybackburgers.com). While a growing market is emerging, establishing robust supply chains, from insect farming to ingredient processing, is essential to scale up production and meet the standards of the food and beverage industry.

Overall, this category illustrates the strongest alignment between nutritional evidence, technological feasibility, and functional positioning. Nevertheless, further optimization is required to standardize ingredient quality, improve flavor neutrality, and establish evidence-based formulation guidelines for beverage applications.

### 5.2. Fermented and Gut Health-Promoting Beverages

Fermented beverages constitute a promising yet still experimental application for insect-derived ingredients. In this category, insects are primarily used to enhance protein quality, support probiotic viability, or contribute fermentable substrates such as chitin-derived compounds [7,32].

Studies on non-alcoholic fermented beverages enriched with cricket protein hydrolysates demonstrate that processing strategies combining enzymatic treatment, ultrasonication, and fermentation can significantly improve protein solubility, digestibility, and peptide bioavailability while maintaining high probiotic counts during storage [7,14,42]. These findings suggest that insect proteins may function not only as nutrient sources but also as protective matrices that enhance probiotic stability under gastrointestinal conditions.

On the other hand, chitin and its derivatives have also been explored as prebiotic components in gut health-oriented beverages. Although native chitin is insoluble, partial hydrolysis into chitosan or chitooligosaccharides can increase fermentability and microbiota-modulating potential [11,33].

A chitin-based beverage can be formulated with ingredients such as ginger, lemon, and fermented apple cider vinegar, resulting in a refreshing and tangy prebiotic drink [33]. Chitin is an insoluble dietary fiber naturally present in the exoskeletons of insects. While it offers prebiotic benefits, its low water solubility poses a challenge for beverage applications. In the human gut, insoluble fibers such as chitin contribute to bowel regularity, fecal bulking, and improved gut motility while also serving as substrates for certain microbial communities.

Dridi et al. [7] and Dridi et al. [14] explored the sensorial and probiotic stability of fermented beverages enriched with house crickets. In the first study, the combination of ultrasound (tUS = 20 min) followed by γ-irradiation (dose = 3 kGy) showed a raised protein solubility of 29.9%, up to 2.6-fold compared to individual or untreated samples; a 94% increase in protein digestibility; and a reduction in high-molecular-weight peptides to just 0.7% of total peptides in cricket-fermented samples [7]. In the second study, results indicated that the addition of cricket protein hydrolysate did not alter texture (score of 7.1) or color (score of 6.7) compared to the control (*p* > 0.05) while maintaining a high concentration of viable probiotics at 8.45 log/mL after 5 months of storage. Additionally, the fermented cricket beverage exhibited a significant amount of low-molecular-weight peptides (<0.26 kDa), which continued to increase during storage, reaching 74.7% compared to 59.9% in the control (*p* < 0.05). Beyond protein improvement, insect fermentation may also enhance the gut-health potential of other insect-derived components, such as chitin. Although chitin is an insoluble dietary fiber, slow fermentation by colonic microbiota can generate short-chain fatty acids and bioactive chitooligosaccharides, supporting intestinal health and microbial diversity. Furthermore, probiotics demonstrated strong resistance to in vitro gastrointestinal conditions, particularly in the cricket-enriched beverage, which maintained an 83% survival rate of viable probiotics (lactic acid bacteria) after 5 months [14]. This protective effect may be attributed to the presence of insect proteins and peptides, which can form matrices or interact with probiotic cells, enhancing their stability and resistance during digestion. These characteristics position these fermented beverages similarly to established market leaders such as kombucha and kefir, not because insect proteins act as probiotics themselves, but because they can serve as protective carriers that support probiotic viability and function.

It is important to note, however, that although proteins themselves are not classified as prebiotics under current definitions, certain peptides derived from enzymatic or microbial processing may exert microbiota-modulating effects and indirectly contribute to gut health through bioactive or fermentable properties. Therefore, insect-based fermented beverages not only improve digestibility and biofunctionality through processing, but also deliver components that contribute to a healthier gut environment. In this sense, probiotic beverages enriched with cricket protein hydrolysates were formulated using fig juice (40%), carob extract (35%), and water (25%), and fermented with a mixed starter culture of *Lactobacillus delbrueckii* subsp. *bulgaricus* and *Streptococcus thermophilus*; the formulation also included cricket protein hydrolysate at 2% (*w*/*v*) to enhance nutritional value and support probiotic viability [42]. In a Wistar rat model, the beverage’s protein quality, assessed by the protein efficiency ratio (PER 1.2–2.0), the net protein ratio (NPR 0.4–1.0), and apparent digestibility (85–94%), was comparable to casein (up to 96%), but superior to non-hydrolyzed cricket proteins. Following this, the research group also reported that the gut microbiota in the fecal samples of Wistar rats was modified after 14 days of feeding with cricket protein or a cricket protein hydrolyzed-enriched fermented probiotic product [50]. Taxonomic results showed no significant differences (*p* > 0.05) in microbial diversity and richness between the groups fed casein (control) and cricket probiotic beverages. The composition of the microbiota was significantly altered when rats were fed with cricket beverages (*p* < 0.05), especially in the families Christensenellaceae, Eggerthellaceae, Peptostreptococcaceae, and Ruminococcaceae, among others. Still, a statistically significant (*p* < 0.05) decrease was observed in the potential pathogenic bacteria of the Enterococcaceae family. Additionally, the protein quality parameters showed that the in vivo digestibility of the cricket protein-hydrolyzed beverage significantly increased (*p* < 0.05), resulting in a PER increase from 1.7 to 2.0 and an NPR increase from 0.4 to 1.0, compared to the cricket protein-not-hydrolyzed beverage [50].

Despite encouraging experimental results, fermented insect-based beverages remain largely confined to laboratory-scale studies. Their transition to commercial products will depend on further validation of health effects, optimization of sensory profiles, and alignment with regulatory definitions of probiotics and prebiotics. However, incorporating these compounds into beverages requires careful control of particle size, dosage, and processing conditions to prevent sedimentation and sensory defects.

### 5.3. Alcoholic Beverages: Beers, Spirits, and Distilled Products

**Figure 2:**
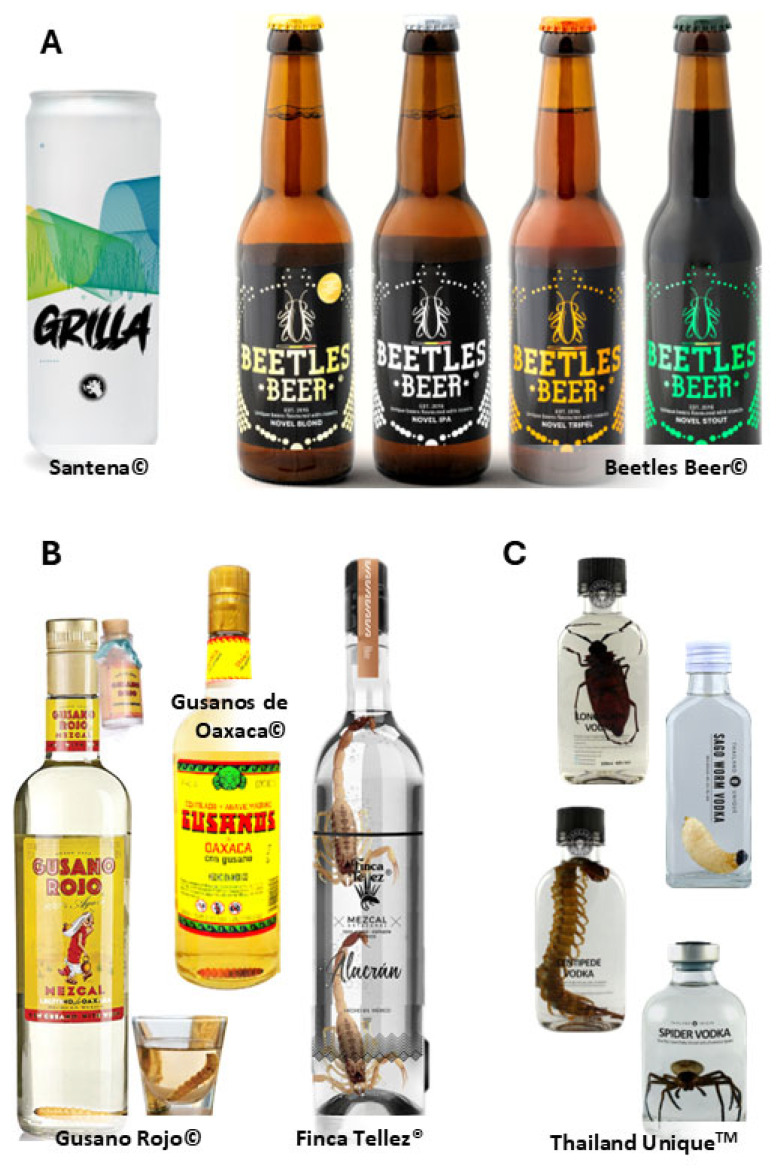
Examples of commercially available alcoholic beverages made from or with insects and other arthropods. (**A**) Beers made with edible insects (https://santena.mx; https://beetlesbeer.com). (**B**) Mexican mezcals with added whole insects and other arthropods (https://www.mezcalreviews.com; https://comercialjp.cl; https://mezcalfincatellez.com.mx). (**C**) Vodka spirits containing edible insects and other arthropods (http://thailandunique.com). Images were adapted from publicly available online sources, including official producer websites, and assembled by the authors for academic and illustrative purposes only. No endorsement of specific brands is intended.

Several innovative breweries have experimented with incorporating edible insects into beer formulations, creating distinctive products within the craft beer market (Figure 2A). Insects such as crickets and mealworms are often used in roasted or powdered form as adjunct ingredients during brewing, where they may contribute nutty or roasted flavor notes, modify mouthfeel, and increase perceived body due to their protein and lipid content. In some cases, replacing small proportions of malt (e.g., ~5%) with roasted insect powder has been reported to produce sensory profiles comparable to those obtained with traditional grains [8,9]. These formulations demonstrate that insect-based beers can achieve high sensory acceptance when brewing parameters and ingredient proportions are carefully controlled.

As an example, in Mexico, Santena© (https://santena.mx), Punto Medio© (https://cerveceriapuntomedio.com), and Cerveceros Arellano© (https://www.cervecerosarellano.com) made a porter beer named “La Grilla” in 2023. According to the creators, replacing 5% of toasted malt with powdered toasted house crickets provides a flavor similar to barley or rye, along with texture, color, and a higher digestible protein content. Another example is Badger Brewery (https://www.badgerbeers.com), a company renowned for its distinctive range of beers, which produces a stout incorporating cricket flour into the brewing process. The crickets are roasted and ground into a fine powder, which is added to the malt during brewing (https://hywelsbiglog.wordpress.com). This stout is notable for its rich, nutty flavor profile, which is enhanced by the addition of crickets. It offers a higher protein content compared to traditional stouts, making it an appealing option for health-conscious consumers. Beetles Beer© (Belgium, https://beetlesbeer.com) released four different beers made with edible insects. While the beer production process itself contributes to product diversity, the inclusion of different insect species further influences sensory characteristics, such as aroma, color, mouthfeel, and flavor complexity, due to the distinct nutritional compositions and fat profiles of each insect. From this lot, the Novel Blond beer won Gold in the European Beer Challenge 2024 (https://europeanbeerchallenge.org) and Bronze in the World Beer Award Belgium 2024 (http://worldbeerawards.com). The other three beers are made with house cricket (*Acheta domesticus*), mealworm (*Tenebrio molitor*)*,* and locust (*Locusta migratoria*) (Novel Ipa©); house cricket and locust (Novel Tripel©); and house cricket and mealworm (Novel Stout©).

In distilled beverages, insects play a primarily symbolic and sensory role. The presence of the insect is closely linked to notions of tradition, authenticity, and ritual, and its contribution to volatile compounds has been analytically demonstrated [18,54]. In these beverages, insects are typically added post-distillation and are not intended to function as nutritional ingredients.

The incorporation of edible insects into distilled and alcoholic beverages, particularly in traditional Mexican spirits such as tequila and mezcal, is a fascinating aspect of culinary innovation (Figure 2B). Mezcal, known as “the worm’s beverage”, is a distilled spirit made from some agave species (*Agave* spp.); the species *Agave salmiana* is preferred [54]. This distillate often includes a single red caterpillar (*Comadia redtenbacheri*) known as “gusano rojo de maguey” (red maguey worm) or “chinicuil” per bottle [18], whose life cycle revolves around this agave plant. The red worms are harvested, sometimes roasted, and then added to the mezcal during the bottling process. The presence of the worm has led to a perception of authenticity and tradition in mezcal production [10]. Some studies in this field have highlighted that the inclusion of red worms is relatively new, dating back to the 1940s and 1950s; however, the reasons are contentious. Authors such as Janzen [46] suggest that the red worm is used as an indicator of alcohol quality and stability, as “if the worm remained intact in the liquid, the alcohol percentage was high enough to preserve it.” Still, others affirm that the red worm is only to reinforce the identity of some mezcal brands such as Gusano Rojo© (Mexico, gusanorojomezcal.com) and Mezcal Monte Albán© (Mexico) [9]. As noted by De León-Rodríguez et al. [54], red worms contribute distinctive volatile compounds to mezcals (i.e., 6,9-pentadecadien-1-ol, 3-hexen-1-ol, 1,8-nonadiene, and 1-dodecine) that are not found in mezcal samples lacking worms, making them appealing to liquor connoisseurs and enthusiasts. These worms are credited with hallucinogenic and aphrodisiac properties, but none of these have been proven.

Although worms are not included in the fermentation process, they are the main distinguishing characteristic of mezcal, giving it an “earthy” flavor and masking unwanted flavors [9]. However, new mezcal formulations are aged in oak barrels, and the worm intensifies their aromatic profiles, as seen in the El Cotijo© (https://mezcalelcortijo.com) and Wahaka© (https://www.wahakamezcal.com) mezcals. According to various alcohol shopping websites such as SoyOaxaca (https://www.soyoaxaca.com) and OaxacaMezcal (https://oaxacamezcal.top), some mezcal brands have recently incorporated giant scorpions (possibly *Hadrurus*, *Hoffmannihadrurus* or *Chihuahuanus* species) to their bottles such as Mezcal Finca Tellez^®^ (https://mezcalfincatellez.com.mx). These e-shopping websites mention that mezcal with scorpion has beneficial properties, such as relieving pain, inflammation, and migraines, as well as being a possible remedy for chronic diseases such as diabetes and arthritis. However, none of these supposed health benefits has been proven.

Agave distillates, unlike tequila and mezcal, which are protected by designation of origin, do not have such legal status; for instance, tequila must be made from *Agave tequilana* var. *azul* and produced within specific regions of Mexico [47]. However, all three types of spirits can include edible insects for various purposes, such as enhancing flavor, symbolizing authenticity or artisanal value, or serving as a marketing element to attract curious consumers. While traditional tequila does not typically include insects, some craft distilleries have begun experimenting with adding edible insects to agave distillates. For example, Gusanos de Oaxaca^®^ is an agave distillate with larvae of white maguey worm or “meocuilín” (*Aegiale hesperiaris*). This insect can be infused into the spirit or used as a garnish. This approach offers a novel twist on classic maguey distillates, enhancing their attractiveness. It appeals to adventurous consumers looking for unique tasting experiences.

Some distilleries are creating insect-infused spirits by steeping edible insects in alcohol. For example, house crickets or yellow mealworms can be added to vodka, allowing their flavors to infuse into the spirit (Figure 2C). For instance, Thailand Unique^TM^ (Thailand, https://www.thailandunique.com) is a brand that offers a wide range of insect-based and arthropod-based products on its menu, such as vodka or vodka infusions that contain whole individuals of Chinese red-headed centipede (*Scolopendra mutilans*), Asian forest scorpions (*Heterometrus longimanus*), giant crab spider (*Heteropoda venatoria*), bamboo worm (*Omphisa fuscidentalis*), Asian longhorn beetle (*Rosenbergia* sp.) or zebra leg tarantula (*Cyriopagopus albostriatus*), sago worm (*Rhynchophorus ferrugineus*), and Chinese armor tail scorpion (*Mesobuthus martensii*). These products stand out due to their innovative use of insects, providing a distinct flavor and sparking conversation, as they offer a new way to consume edible insects and other arthropods commonly included in traditional Thai menus [55].

Interestingly, bartenders and mixologists around the world are increasingly incorporating edible insects into cocktails, either as decoration or blended into the drinks. For example, Sangrita infusion by Santa Prisca (Mexico) includes milpa grasshoppers (*Sphenarium purpurascens*) in its formulation, which is used to create mixes with tequila and mezcal [51]. Additionally, milpa grasshopper salt (essentially grasshopper powder mixed with an equal amount of salt) can be used to rim glasses, or insect powders can be mixed into cocktails for added flavor [19].

Occasionally, red maguey worms (*Comadia redtenbacheri*) and scorpions (*Centruroides* spp.) are used as substitutes in milpa grasshopper salt preparations. Still, powdered chili peppers and other condiments can also be included [56]. This trend introduces a creative and experiential element to cocktail culture, appealing to consumers seeking unique and sustainable drinking options. The incorporation of edible insects in spirits and beers reflects a growing interest in innovation and product differentiation within the food and beverage industry. In distilled spirits, insects are typically added at the end of the process, floating in the bottle as optional edible components and serving symbolic, flavoring, or marketing purposes. In contrast, craft beers may incorporate insects as fermentable ingredients, such as roasted or ground cricket flour, which directly contributes to the sensory profile and nutritional composition. While this opens new opportunities for microbial fermentations using insect-derived substrates in other beverage types, it also presents challenges, including potential impacts on flavor stability, allergen management, regulatory approval, and consumer acceptance. Addressing these issues will be key to expanding the use of edible insects in alcoholic and functional beverage markets.

### 5.4. Teas and Traditional Beverages

Insect-based teas represent a distinct category rooted in traditional practices, particularly in parts of Asia [57]. In regions such as the Changjiang River basin in China, ethnic minority groups have long prepared teas from insect feces, notably from species such as *Pyralis farinalis*, *Hydrillodes morosa*, and *Aglossa dimidiata*. One such tea, known as “Chong Cha,” reflects deep-rooted cultural traditions despite its high production cost and limited accessibility [48,58].

Other insect feces teas are produced through a unique process that involves insects that feed on specific plants. Production methods vary, but they generally follow similar household protocols. For example, to produce “Chengbu Miao insect tea”, the Miao people collect leaves from different plants in late spring and early summer. Some leaves are air-dried for daily consumption, while others are boiled, air-dried, and then sprayed with rice-washed water to attract insects. The leaves are placed in a ventilated area, and the rice-washed water is reapplied every few weeks to encourage insect egg-laying. Once the larvae hatch and consume the leaves, their feces are collected, which is the final product known as insect tea. This process can take nearly a year to yield just 1 kg of insect tea from 10 kg of leaves, making it a labor-intensive endeavor [49]. Various types of insects are used to produce this insect tea, including *Aglossa dimidiata* fed on *Malus sieboldii* (producing Sanye insect tea), *Hydrillodes morosus* fed on *Platycarya strobilacea* (produce Hua-Xiang insect tea) and *Aglossa dimidiata* fed on *Litsea coreana* (produce Hawk insect tea) [49]. The diversity of insect species and the plants on which they feed contributes to the broad range of insect teas available, each of which is characterised by unique flavour attributes and potential health benefits. However, a taxonomic study on the bacteria contained in insect fecal samples after the tea-making process revealed that the microbiological risks associated with consuming these samples in traditional ways could pose a potential health risk to consumers [48].

Recent research has identified a broad spectrum of bioactive compounds in insect teas, including high concentrations of flavonoids (quercetin: 3.2–7.6 mg/g; kaempferol: 1.9–4.5 mg/g; rutin: 2.4–6.8 mg/g) and phenolic acids (gallic acid: up to 2.7 mg/g; chlorogenic acid: up to 1.9 mg/g), along with polysaccharides, alkaloids, saponins, and essential minerals like zinc, iron, and selenium [58]. These compounds exhibit antioxidant, anti-inflammatory, hypoglycemic, and hepatoprotective effects, among others. Rodent studies suggest that insect tea consumption can reduce oxidative stress, improve blood glucose and lipid profiles, and support gut microbiota balance. These benefits are likely enhanced by the fermentation process occurring in the insect gut, which may improve compound bioavailability.

Commercial versions of these teas are beginning to emerge. For example, Thailand Unique™ (Thailand, thailandunique.com) markets “Bugapoop Worm Poo” and “Frass for Tea Bags,” which are derived from *Bombyx mori* and *Andraca* sp., and promoted as being rich in amino acids, vitamins, and minerals. However, despite their traditional and nutritional appeal, concerns remain. As with Chinese traditional teas, microbiological risks in commercially produced products remain a key safety challenge [48]. The complex composition of insect frass, including residual proteins, plant-derived metabolites, and microbial content, offers both potential and uncertainty. Additionally, sensory limitations such as bitterness or earthy aromas could hinder acceptance in markets unfamiliar with insect-derived products.

Future directions could include blending insect products with medicinal plants to enhance both taste and health effects, improving processing methods to enhance microbiological safety, and characterizing the nutritional content. To legitimize insect teas as functional beverages globally, further research is needed to validate health claims, enhance sensory properties, and ensure safe and standardized production practices.

Additionally, traditional cereal-based beverages also illustrate the feasibility of incorporating edible insects without compromising sensory acceptance. A notable example is the enrichment of “pinole”—a toasted maize-based product traditionally consumed as an “atole”—with chapulin (*Sphenarium* spp.) flour. Experimental formulations containing up to 50% insect flour showed no significant differences in consumer preference compared to conventional formulations while achieving a 365% increase in protein content and substantial improvements in essential amino acid profiles [58]. These results highlight how culturally embedded beverage formats can accommodate high levels of insect inclusion when sensory expectations are aligned with tradition.

### 5.5. Insect-Based Milk Analogues

A milk analog is a product that mimics the properties and functions of traditional milk, made from non-dairy sources, such as plant-based proteins [59]. These products are designed to replicate the taste, texture, nutritional profile, and functional characteristics of milk, often tailored to specific dietary needs or preferences [60,61]. Almond, soy, rice, oat, coconut, cashew, hemp, and pea milk are examples of milk analogs available in the market [60]. These milk analogs meet various dietary preferences and restrictions, providing consumers with a range of options to choose from based on taste, nutrition, and sustainability [61]. Interestingly, milk analogues can also be made from edible insects. For example, Tello et al. [62] developed an insect-based milk analogue by mixing ground meal-worm (*Tenebrio molitor*) with water, ascorbic acid, extracted fat, and sunflower lecithin, followed by filtration and homogenization to achieve a creamy texture and desired nutritional composition. This “mealworm milk” prototype contains 1.19% crude protein and 5.76% fat; it has a lower protein content than traditional milk but exhibits similar sensory properties to bovine milk, such as a creamy texture and a beige color.

The Pacific beetle cockroach (*Diploptera punctata*) produces a nutrient-rich secretion that has attracted scientific attention due to its unusual biochemical composition [63,64]. This secretion, sometimes referred to in the literature as “cockroach milk,” is produced to nourish developing embryos in this viviparous species. It contains proteins, carbohydrates, and lipids including oleic acid, conjugated linoleic acid, omega-3 fatty acids, and several vitamins [65]. A distinctive feature of this secretion is the presence of protein crystals known as “Lili-Mip,” which contain a mixture of proteins, lipids, and carbohydrates that function as a concentrated nutrient source for developing embryos [66,67,68]. These crystals release amino acids, fatty acids, and peptides during digestion, reflecting a complex biochemical structure [65,69].

Despite the biochemical interest surrounding this secretion, its direct application in food systems or beverage formulations remains largely theoretical and faces substantial technical and ethical challenges. Nevertheless, the study of such insect-derived secretions has contributed to broader discussions on unconventional nutrient sources and may inspire future research on novel ingredients for specialized food or beverage formulations. Similar secretory systems with nutritionally relevant compounds have been reported in other insects and arthropods, suggesting that these biological mechanisms may represent a potential area for further investigation [70].

Additionally, the production of insect milk analogues has a lower environmental impact compared to bovine milk, with a significantly reduced carbon footprint and resource usage, which aligns with the growing demand for sustainable food alternatives [71]. In particular, harvesting this substance directly from insects poses substantial ethical, biological, and logistical challenges for large-scale production. As a result, future efforts may prioritize the biotechnological replication of cockroach milk components through microbial fermentation or synthetic biology, enabling controlled, ethical, and scalable production. Rather than aiming for bulk production for direct consumption, small quantities of this nutrient-rich secretion could be incorporated as bioactive ingredients in specialized functional formulations.

At present, insect-based milk analogues remain at a proof-of-concept stage. Their relevance lies primarily in expanding the conceptual boundaries of alternative dairy research rather than in near-term commercial application. Although the direct use of this secretion in food systems remains largely theoretical and faces significant practical and ethical challenges, it has been discussed as a potential source of bioactive compounds that could inspire future ingredient development for specialized food or beverage formulations.

### 5.6. Critical Synthesis of the Current State

Across all beverage categories, a clear pattern emerges: insect-based beverages are not a single product class but a spectrum of applications with distinct objectives, technological requirements, and consumer expectations. Functional beverages prioritize invisibility, stability, and nutritional efficacy, while experiential beverages emphasize visibility, flavor, and cultural meaning.

**Figure 3:**
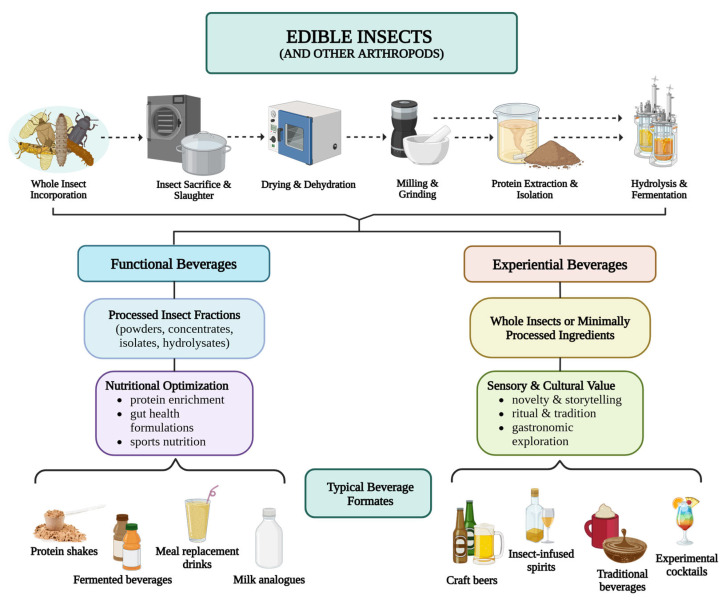
Conceptual pathways for the development of insect-based beverages.

The diversity of examples discussed throughout this section can be more clearly interpreted when viewed through the conceptual pathways proposed in this review. As illustrated in Figure 3, the incorporation of insect-derived ingredients into beverages follows different formulation logics that reflect distinct product design priorities. In functional beverages, insects are typically processed into technologically compatible fractions—such as powders, protein concentrates, isolates, or hydrolysates—that enable their integration into liquid matrices to deliver measurable nutritional or physiological benefits. By contrast, experiential beverages often incorporate whole insects or minimally processed ingredients whose value lies primarily in their sensory contributions, symbolic meanings, or narrative appeals within culinary and cultural contexts. Importantly, these pathways should not be interpreted as rigid categories but rather as analytical orientations within a broader continuum of product development strategies. Some formulations may combine functional objectives with experiential elements, for example, by embedding insect-derived nutrients within culturally meaningful beverage formats. The conceptual framework summarized in Figure 3 therefore provides a useful lens for interpreting the heterogeneous landscape of insect-based beverages and for identifying future opportunities at the intersection of technology, gastronomy, and consumer perception.

Failure to differentiate between these approaches has contributed to conceptual confusion in both academic and commercial discourse. A more nuanced understanding of application-specific constraints is therefore essential for guiding future research, formulation strategies, and market positioning.

## 6. Consumer Perception and Acceptance of Insect-Based Beverages

Consumer acceptance represents one of the most significant barriers to the development and commercialization of insect-based beverages. Although edible insects are increasingly recognized for their sustainability and nutritional potential, their integration into beverages introduces perceptional dynamics that differ substantially from those associated with solid foods. Factors such as visual appearance, clarity, mouthfeel, aroma release, and consumption context play a disproportionately important role in shaping consumer responses to liquid products.

Most consumer studies on edible insects have focused on solid foods, such as snacks, bakery products, or meat analogues, and their conclusions are often extrapolated to beverages without sufficient justification [4,72]. However, beverages differ fundamentally from solid foods in terms of sensory expectations and consumption rituals. Even minor changes in turbidity, color, or aroma may be perceived as quality defects rather than indicators of novelty or functionality, particularly in categories such as juices, isotonic drinks, or fermented beverages.

Within the functional beverage pathway, consumer acceptance is closely linked to familiarity, invisibility of the insect ingredient, and alignment with health-related expectations. Studies indicate that powdered or refined insect ingredients are more readily accepted than whole insects, as they minimize visual cues associated with disgust and neophobia [17,73]. In beverages, this preference is even more pronounced, as consumers typically expect homogeneity and smooth texture. Formulations that rely on protein isolates, hydrolysates, or highly processed fractions are therefore more likely to succeed in this segment.

Research has shown that familiarity with insect-based products and exposure to information about their environmental and nutritional benefits could help mitigate this resistance [17,73,74]. Furthermore, insect presentation plays a significant role in consumer acceptance. For example, evidence from traditional beverages such as “chucula” fortified with 4.6% cricket flour showed an 8% reduction in purchase intention, but with a general acceptance of 74% among the panelists [45]. In another study, Montoro et al. [45] found that incorporating cricket flour into chucula significantly enhanced its nutritional profile, particularly its protein content. For instance, sample formulations with varying percentages of cricket flour showed crude protein values (16.4% to 17.4%), depending on the specific formulation. The study also reported a water absorption index ranging from 2.37 to 2.7, indicating the extent to which the samples absorbed water, while the solubility index varied from 12.7% to 13.4%. These findings suggest that adding cricket flour increases the protein content and affects the physicochemical properties of chucula, making it a more nutritious beverage without compromising its traditional characteristics [45].

The evidence also suggests that ground or powdered insect ingredients are generally more widely accepted than whole insects, as they can be incorporated into beverages without significantly affecting their appearance or texture [4,72]. In the case of chapulin-enriched “atole”, even formulations with pronounced insect flavor were positively evaluated, indicating that visibility and intensity of insect-derived notes do not necessarily reduce acceptance when embedded within culturally recognized products [58].

Taste and aroma also play a critical role. Although insect-derived ingredients may exhibit nutty or roasted notes that are acceptable—or even desirable—in some applications, they may conflict with consumer expectations in fruit-based or neutral beverages [8]. Advances in deodorization, lipid removal, controlled fermentation, and flavor-masking technologies have been shown to mitigate these challenges and improve acceptance [2,43]. Importantly, acceptance in functional beverages is often driven less by novelty and more by perceived efficacy, convenience, and trust in safety and regulation.

Despite these technological strategies, sensory limitations remain a significant barrier to the wider adoption of insect-based beverages. Several studies report that insect-derived ingredients may introduce earthy, roasted, or fermented flavor notes that do not always align with consumer expectations for beverages, particularly in fruit-based or lightly flavored drinks. In addition, darker coloration and increased turbidity resulting from insect powders can negatively affect visual acceptance in beverage categories where clarity and brightness are typically associated with quality. In some experimental formulations, higher inclusion levels of insect ingredients have led to reduced purchase intention or lower sensory scores, underscoring the need for careful formulation strategies and flavor integration.

In contrast, within the experiential and storytelling-driven pathway, consumer acceptance follows a different logic. Here, visibility of the insect ingredient, sensory distinctiveness, and cultural framing may enhance rather than hinder acceptance. Beverages such as mezcal containing red maguey worms or craft beers brewed with insects are consumed within specific ritualized contexts, where novelty, authenticity, and narrative value are central to the experience [9,75]. In these cases, insects are not perceived as contaminants but as integral components of the product’s identity.

Acceptance in this pathway is strongly influenced by context, including the place of consumption, social setting, and symbolic associations. Theories of food practice emphasize that novel foods are more readily accepted when embedded within coherent cultural narratives, supported by culinary expertise, and consumed in socially meaningful environments [20]. Rather than focusing on individual attitudes alone, these perspectives highlight the importance of chefs, bartenders, rituals, and storytelling in shaping consumer experiences with insect-based beverages.

Notably, these two acceptance mechanisms—functional invisibility and experiential visibility—are often conflated in both academic discourse and product development. Attempts to market insect-based beverages simultaneously as health-optimized functional products and as novelty-driven experiential items may result in ambiguous messaging and reduced acceptance. Clear alignment between product design, sensory profile, and value proposition is therefore essential.

Finally, trust in regulation, labeling, and safety plays a critical role across both pathways. Transparent labeling, including the use of scientific species names, clear information on processing methods, and compliance with novel food regulations, contributes to consumer confidence and reduces perceived risk [17]. In the absence of such clarity, even well-formulated products may face resistance due to uncertainty rather than sensory rejection.

In summary, consumer acceptance of insect-based beverages cannot be understood through a single explanatory framework. Functional beverages require technological strategies that minimize sensory disruption and emphasize health efficacy, while experiential beverages depend on cultural embedding, storytelling, and sensory engagement. Recognizing and respecting these distinct acceptance pathways is a prerequisite for advancing insect-based beverages beyond niche markets and into broader food systems.

## 7. Challenges and Future Directions

Although entomophagy is widely practiced across many regions of the world—particularly in tropical areas of Africa, Asia, and Latin America—the documented use of insects specifically in beverage formulations remains geographically uneven in the scientific and commercial literature. Much of the currently available information originates from Western product development contexts or from well-documented traditional cases such as those reported in Mexico and parts of Asia. This pattern likely reflects disparities in research attention and product commercialization rather than the true global distribution of entomophagy practices. In many cultures with long-standing traditions of insect consumption, insects are primarily incorporated into solid foods rather than beverages, which may partly explain their limited representation in beverage-focused studies. Consequently, the apparent geographic imbalance observed in the literature highlights an important knowledge gap and suggests that future research may reveal additional culturally embedded beverage applications that remain undocumented.

Despite the growing scientific and commercial interest in insect-based beverages, several interconnected challenges continue to limit their development beyond niche applications. These challenges span technological, regulatory, supply chain, and perceptional dimensions and differ markedly depending on whether beverages are positioned within functional or experiential pathways.

One of the most fundamental constraints is the lack of ingredient specialization tailored specifically to beverage systems. Although edible insects are increasingly produced for food and feed, most commercially available ingredients are generic flours or minimally processed powders. Such ingredients are often unsuitable for liquid formulations, which require precise control over solubility, particle size, stability, and sensory neutrality. In contrast to established plant and dairy protein industries, where protein isolates, hydrolysates, and functional fractions are produced for specific applications, insect-based ingredients for beverages remain underdeveloped [3,16].

Technological innovation is therefore a priority. Processing strategies such as enzymatic hydrolysis, fermentation, ultrasonication, membrane fractionation, and deodorization have demonstrated significant potential to improve the techno-functional and sensory performance of insect-derived ingredients [6,7,39]. However, most studies remain at laboratory scale, and their industrial scalability, economic feasibility, and environmental trade-offs require further evaluation. In addition, formulation guidelines specific to beverage matrices—such as optimal protein concentrations, pH ranges, and stability thresholds—are still lacking.

Another important challenge relates to allergen management in products containing insect-derived ingredients. Certain insect proteins, such as tropomyosin and arginine kinase, may cross-react with allergens found in crustaceans and mites, which raises potential safety concerns for sensitive consumers. Consequently, appropriate risk assessment, transparent allergen labeling, and clear consumer guidance are essential to ensure product safety when developing insect-based beverages. In addition, manufacturing practices must account for potential cross-contamination during processing and packaging, particularly when insect-derived ingredients are handled in facilities that also process conventional food products. Establishing appropriate precautionary labeling and considering allergen thresholds for sensitive individuals remain important regulatory and research challenges in this emerging sector.

Regulatory uncertainty represents another critical barrier. In many regions, insect-based ingredients are regulated as novel foods, with conditions of use that depend on insect species, processing methods, and food categories [17,76]. In the European Union, edible insects are regulated under the Novel Food framework, and their use is authorized through specific Implementing Regulations that define permitted species, processing forms, and food categories [77]. These authorizations may include maximum inclusion levels for particular food applications, including certain beverage categories. However, these limits vary by insect species and authorized product form (e.g., frozen, dried, or powdered) and should therefore be interpreted within the context of the specific regulatory approval rather than as universal thresholds. According to Mancini et al. [17], the maximum allowed amount of *Locusta migratoria* in beer, beer-like beverages, unsweetened spirits, and liqueurs is 2 g per 100 g, while for *Tenebrio molitor* and *Acheta domesticus*, it is 1 g per 100 g, regardless of whether in dried, frozen, or powdered form. Clear, harmonized legislation and transparent labeling standards (e.g., scientific names rather than vague common names) will be essential for consumer trust and industry scalability [77]. In addition to the European Union’s Novel Food framework, regulatory uncertainty in other markets—such as the lack of widely recognized GRAS status for most insect-derived ingredients in the United States—may further complicate the commercialization of insect-based beverages.

However, these authorizations do not constitute universal quantitative limits applicable across all beverage types, and their interpretation requires careful consideration of the specific product formulation and processing context. Oversimplified representations of regulatory thresholds may therefore mislead both researchers and product developers. Clearer guidance, harmonized labeling practices, and transparent communication—particularly regarding species identification and processing forms—are essential to support responsible innovation and consumer trust.

Supply chain limitations further constrain the sector. While insect farming has expanded, consistent access to high-quality, food-grade insect ingredients with reproducible functional properties remains limited. Beverage applications demand stricter quality control than many solid foods, particularly regarding microbial safety, oxidation stability, and batch-to-batch consistency. Strengthening links between insect producers, ingredient processors, and beverage manufacturers will be essential to support scale-up and standardization [78].

From a market perspective, a persistent challenge lies in aligning product design with consumer expectations. As highlighted throughout this review, insect-based beverages operate within two distinct acceptance pathways. Functional beverages require invisibility, sensory neutrality, and evidence-based health positioning, whereas experiential beverages rely on visibility, narrative coherence, and cultural embedding. Attempts to merge these logics without clear prioritization may result in ambiguous products that fail to resonate with either audience. Future product development should therefore adopt application-specific strategies that explicitly recognize these differences.

As noted by the American Craft Beer portal (https://www.americancraftbeer.com), some sectors still label insect-based beers under “Bad Ideas In Brewing” (https://www.beerandbrewing.com), showing lingering skepticism. Integrating insect-derived ingredients with other familiar protein sources (e.g., plant or dairy proteins) and functional co-ingredients (e.g., cocoa, fruit extracts, probiotics) may help improve acceptability and facilitate market entry.

A thorough investigation of Patentscope (https://www.wipo.int/en/web/patentscope), the database of the World Intellectual Property Organization (https://www.wipo.int), revealed that there are currently no patents concerning the formulation, development, marketing, or production of beverages or beverage ingredients derived from insects. The bibliography includes only one report of a patent for a protein powder created from an unspecified insect, developed by the Mississippi State University Insect Rearing Centre in collaboration with Neptune Industries Inc. (USA), and registered under the name Ento–Protein™ [79]. Although the patent holders claim that the product possesses suitable amino acid profiles, thermal stability, solubility, gelling, foaming, and emulsifying properties for beverage formulations, the specific values for these characteristics have not been disclosed. As a result, the exact suitability of this product for formulating particular beverages remains unclear. The above-mentioned indicates a significant opportunity for innovation and research in this field, suggesting that future developments could lead to the creation of unique insect-based beverages that capitalize on their nutritional and health benefits.

Finally, it is important to highlight that with increasing consumer demand and projected global market growth, expected to reach up to US$18 billion by 2050 [80,81], this sector represents a significant opportunity for innovation. Finally, significant knowledge gaps remain regarding effective dosages, long-term health effects, and bioavailability of insect-derived components when delivered through beverages. While human intervention studies support the nutritional quality of insect proteins, evidence linking insect-based beverages to specific health outcomes—such as improved gut health, immune modulation, or performance enhancement—remains limited. Addressing these gaps will require well-designed human studies, standardized formulations, and interdisciplinary collaboration across food science, nutrition, sensory science, and social research.

## 8. Conclusions

Insect-based beverages represent a highly diverse, still-emerging category at the intersection of food science, beverage technology, and food culture. This work highlights that edible insects cannot be treated as a uniform ingredient across beverage applications, as their nutritional relevance, techno-functional performance, sensory impact, and cultural meaning vary substantially depending on formulation strategy and consumption context. By distinguishing between functional beverages and experiential, storytelling-driven beverages, this review provides a conceptual framework that clarifies much of the apparent inconsistency in current research and market discourse. Functional applications prioritize nutritional efficacy, solubility, stability, and sensory neutrality, whereas experiential beverages emphasize flavor, ritual, symbolism, and cultural identity. Recognizing this distinction is essential for realistic product development, appropriate regulatory interpretation, and meaningful consumer engagement.

The available evidence indicates that insect-derived proteins, peptides, and chitin-related compounds hold genuine potential for beverage applications when appropriately processed and formulated. However, the transition from experimental concepts to scalable products will depend on the development of specialized ingredients, validated processing technologies, and application-specific formulation guidelines. At the same time, traditional, culturally embedded beverages illustrate that insects can also serve as culinary agents whose value extends beyond nutrition. Ultimately, the future of insect-based beverages will depend not only on technological optimization and a clear regulatory framework but also on the deliberate integration of functionality with sensory quality, cultural narratives, and consumer trust. Rather than seeking universal solutions, progress in this field will require differentiated strategies that respect the complexity of beverage systems and the diverse ways in which insects can contribute to them.

## Figures and Tables

**Table 1:** Properties of insects used as ingredients for beverage formulations.

Order	Scientific Name	Common Name	Insect-Based Ingredient	Type of Beverages	Conceptual Pathway	Properties and Characteristics	Sources
Coleoptera	*Alphitobius diaperinus*	Lesser mealworm	Insect powder	Protein shakes	Functional	Post-prandial BCAA and leucine levels similar to whey and soy protein intake; high and quick amino acid absorption; boost muscle protein synthesis	[27,28]
	*Tenebrio molitor*	Yellow mealworm	Insect powder	Beers, milk analog	Functional	Unique and distinct flavor; nutritional improvement	https://beetlesbeer.com
	*Rosenbergia* sp.	Asian longhorn beetle	Whole insect	Vodka or vodka infusion	Experiential	Unique and distinct flavor	https://www.thailandunique.com
	*Rhynchophorus ferrugineus*	Sago worm	Whole insect	Vodka or vodka infusion	Experiential	Unique and distinct flavor	https://www.thailandunique.com
Lepidoptera	*Comadia redtenbacheri*	Red maguey worm	Whole insect; insect powder	Mezcal; salty powder	Experiential	Enhanced sensory attributes, masking not desirable flavors	[46]
	*Aegiale hesperiaris*	White maguey worm	Whole insect	Agave distillates	Experiential	flavor enhancer; nutritional content enhancer	[47]
	*Pyralis farinalis*	Meal moth	Insect feces	Chong cha tea	Functional/Experiential	Flavor: several health benefits (not proven)	[48]
	*Aglossa dimidiatus*	Moth	Insect feces	Sanye insect tea; Hawk insect tea	Functional/Experiential	Unique flavor and potential health benefits	[49]
	*Hydrillodes morosus*	Moth	Insect feces	Hua-Xiang insect tea	Functional/Experiential	Unique flavor and potential health benefits	[49]
	*Bombyx mori*	Silkworm	Insect feces	Bugapoop Worm Poo and Frass for Tea Bags	Functional/Experiential	Not specified	https://www.thailandunique.com
	*Andraca* sp.	Endromid moth caterpillar	Insect feces	Bugapoop Worm Poo and Frass for Tea Bags	Functional/Experiential	Not specified	https://www.thailandunique.com
	*Omphisa fuscidentalis*	Bamboo worm	Whole insect	Vodka or vodka infusion	Experiential	Unique and distinct flavor	https://www.thailandunique.com
Orthoptera	*Acheta domesticus*	House cricket	Insect Powder, Protein Concentrates and Protein Isolates	Protein shakes, protein smoothies, meal replacements, fermented protein hydrolysates, beers	Functional	Fast post-prandial amino acid recovery and normal appetite hormones, feelings of hunger, and voluntary energy intake among young men; high protein solubility and in vitro digestibility; color and texture enhancement; high nutritional quality parameters; high in vivo digestibility; decreasing potential pathologic bacteria; high probiotic promotion	[7,14,29,42,50] https://beetlesbeer.com; https://santena.mx
	*Locusta migratoria*	Migratory locust	Insect powder	Beers	Functional	Enhanced sensory attributes	https://beetlesbeer.com
	*Sphenarium purpurascens*	Milpa grasshopper	Insect powder	Infusion, salty powder	Functional/Experiential	Unique flavor and texture	[19,51]
	*Sphenarium* spp.	Grasshoppers	Insect powder	Pinole, atole	Functional	>365–480% protein with addition 40–50%; higher Lys, Leu and Val content; techno-functionally compatible with maize flour; good public acceptance in 50:50 ratio;	[52]

## Data Availability

No new data were created or analyzed in this study. Data sharing is not applicable to this article.
